# Neoadjuvant Chemoradiation for Rectal Cancer Achieves Satisfactory Tumour Regression and Local Recurrence – Result of a Dedicated Multi-disciplinary Approach from a South Asian Centre

**DOI:** 10.1186/s12885-023-10769-7

**Published:** 2023-05-04

**Authors:** Raeed Deen, Dileepa S Ediriweera, Suchintha Thillakaratne, Janaki Hewavissenthi, Sumudu K Kumarage, Pramodh C. Chandrasinghe

**Affiliations:** 1 Department of Surgery, Wollongong Hospital, Wollongong, NSW Australia; 2 Health Data Science Unit, Faculty of Medicine, University of Kelaniya, Kelaniya, Sri Lanka; 3 Department of Surgery, University of Kelaniya, Kelaniya, Sri Lanka; 4 Department of Pathology, Faculty of Medicine, University of Kelaniya, Kelaniya, Sri Lanka; 5 Department of Surgery, Faculty of Medicine, University of Kelaniya, Kelaniya, Sri Lanka; 6 The Department of Surgery, University of Kelaniya and North Colombo Teaching Hospital, Ragama, Sri Lanka

**Keywords:** Rectal cancer, Chemoradiation, Tumour regression, Local recurrence, Lymph node harvest

## Abstract

**Background:**

Pre-operative long-course chemoradiotherapy (CRT) for rectal cancer has resulted in improvement in rates of restorative rectal resection and local recurrence by inducing tumour downstaging and downsizing. Total mesorectal excision (TME) is a standardised surgical technique of low anterior resection aimed at the prevention of local tumour recurrence. The purpose of this study was to evaluate tumour response following CRT in a standardised group of patients with rectal cancer.

**Methods:**

One hundred and thirty-one patients (79 male; 52 female, median age 57; interquartile range 47–62 years) of 153 with rectal cancer who underwent pre-operative long-course CRT were treated by standardised open low anterior resection at a median of 10 weeks post-CRT. Sixteen of 131 (12%) were 70 years or older. Median follow-up at the time of analysis was 15 months (interquartile range 6–45 months). Pathology reports were analysed based on AJCC-UICC classification using the TNM system. Data recorded were overall/subgrades of tumour regression; good, moderate or poor, lymph node harvest, local recurrence, disease-free and overall survival using standard statistical methods.

**Results:**

78% showed tumour regression post-CRT; 43% displayed good tumour regression/response while 22% had poor tumour regression/response. All patients had a pre-operative T-stage of either T3 or T4. Post-operation, good responders had a median T stage of T2 vs. T3 in poor responders (P = 0.0002). Overall, the median lymph node harvest was < 12. There was no difference in the number of nodes harvested in good vs. poor responders (Good/moderate-6 nodes vs. Poor- 8; P = 0.31). Good responders tended to have a lesser number of malignant nodes vs. poor responders (P = 0.31). Overall, local recurrence was 6.8% and the anal sphincter preservation rate was 89%. Predicted 5-year disease-free and overall survival were similar between good and poor responders.

**Conclusion:**

Long-course CRT resulted in satisfactory tumour regression and enabled consideration for safe, sphincter-saving resection in rectal cancer. A dedicated multi-disciplinary team approach achieved a global benchmark for local recurrence in a resource-limited setting.

## Introduction

The landscape of treatment for rectal cancer has changed considerably since abdomino-perineal resection (APER) for rectal cancer was first described [1]. Following APER, local recurrence of cancer was as much as 36% [2]. The concept of total mesorectal excision (TME) was popularised in the 1990s [3], where it was shown that removal of the rectum with cancer within its in-tact mesorectum combined with restoration of intestinal continuity, reduced the rate of local recurrence and offered patients life without a permanent stoma. Reduction in local recurrence of cancer was made possible by the use of sharp dissection of the rectum in the presacral plane and by the preservation of an intact mesorectal envelope [3]. Further study of pathology specimens of rectal cancer shed light on the importance of the microscopy of the circumferential resection margin (CRM), where it was shown that if a 2mm microscopic tumour-free margin was achieved at the CRM, local recurrence of rectal cancer reduced from 16–4% [4]. Subsequently, with improved and detailed methods of pre-operative imaging, specifically magnetic resonance imaging (MRI), it was found that the advancing rectal cancer breached the safe CRM in some patients [5, 6]. Irrespective of sharp dissection and TME, the presence of tumour within 2mm of the CRM in these patients increased their risk of local recurrence. These patients were identified as a group that would benefit from neoadjuvant therapy that enabled “pushing back” tumour from its CRM, which enabled the delivery of safe tumour-free margins with the potential for further reduction of local recurrence [7, 8]. This led to the development of pre-operative chemoradiation for rectal cancer, which when combined with sharp dissection TME, resulted in a reduction in local recurrence to its currently acceptable rate of less than 10% [7–9].

The advent of pre-operative chemoradiotherapy (CRT) and now, total neoadjuvant therapy has significantly improved the outcomes of rectal cancer by controlling local recurrence [8, 10], increasing the rate of restoration of intestinal continuity [8] and, in a small proportion, enabling organ preservation following a pathological complete response [10]. Pre-operative CRT is considered the standard of care for locally advanced mid and low rectal adenocarcinoma [11–13]. The histological response of a tumour to CRT, known as tumour regression (TRG), is described using a variety of numerical scales [14]. TRG is characterised by the reduction in the depth of tumour invasion, cytological alterations and stromal reactions such as fibrosis and the formation of mucin pools [14]. Although there is evidence to suggest that pre-operative CRT for rectal cancer might have a significant impact on local disease control, by causing down-staging of a tumour and offering a tumour-free margin of resection, there is no convincing data that show improvement in overall survival [8]. Latterly, however, total neoadjuvant therapy protocols have shown promise and may have the added advantage of prolonging disease-free survival and overall survival in patients with rectal cancer [15, 16]. Additionally, it is recommended that rectal cancer tissue is evaluated for genomic mutations [17]. These add value in prognosis and in guiding treatment with biological agents and novel use of immunotherapy in metastatic rectal cancer that shows progression despite the use of conventional first and second-line treatment [18]. Mutation of RAS protein, microsatellite instability (MSI) and deficiency in mismatch repair protein (dMMR) are such examples, which are associated with oncological aggression [17, 18]. Testing for genomic mutations may be performed upon formalin-fixed tumour tissue or in peripheral blood (liquid biopsy) – the former is preferred over the latter because of the low concentration of genomic antigen in peripheral blood.

There is a paucity of data from South Asia about regression rates of rectal cancer following pre-operative CRT and there is a lack of published data regarding the performance of South Asian colorectal teams that undertake treatment of rectal cancer. The primary aim of this study was to describe the degree of tumour regression with percentage response in a group of Sri Lankan patients with locally advanced rectal cancer, who underwent pre-operative long-course CRT before surgery for low and mid rectal cancer by a dedicated multidisciplinary colorectal cancer team. Secondarily, we aimed to evaluate local recurrence rate, disease-free and overall survival in these patients.

## Materials and methods

### Patient selection and pre-operative work-up

From March 2000 to June 2014, all patients diagnosed with locally advanced rectal cancer based on pelvic MRI and/or CT estimation of tumour extension to either threaten or involve the mesorectal margin [19], were treated with pre-operative long-course CRT followed by TME at the North Colombo Teaching Hospital. Pre-operative work-up of patients included clinical examination with digital rectal examination, assessment of the inferior margin of tumour from the anal verge, serum carcinoembryonic antigen (CEA) level, colonoscopy and biopsy, contrast-enhanced CT scan of the chest, abdomen and pelvis, and MRI of the rectal cancer and pelvis as previously described in detail [20]. Staging of cancer was performed according to the American Joint Committee on Cancer (AJCC) TNM classification [21].

### Pre-operative chemoradiation protocol

Patients received pre-operative CRT at the National Cancer Institute, Maharagama (CIM), under the supervision of a radiation oncologist. Pre-operative CRT comprised radiotherapy (5040cGy) to the true pelvis, which was delivered in 25 fractions combined with cyclical 5-fluorouracil. Following CRT, all patients were assessed fortnightly and underwent one of the following - low or extended low anterior resection with TME of rectal cancer at a median of 10 weeks (range 8–11 weeks) after neoadjuvant therapy, APER, transanal resection of residual tumour or a watch-and-wait protocol. The latter was based on pre-operative clinical, endoscopic and image evaluation one week before surgery.

### Surgical technique and pathology assessment

Surgery was performed by a team of dedicated trained colorectal surgeons at the Northern Colombo Teaching Hospital and surgical specimens were analysed in the Department of Pathology, Faculty of Medicine, Kelaniya, by a colorectal pathologist. Briefly, open surgical operation was performed under general anaesthesia with patients lying supine on the operating table and lower limbs elevated on Allen stirrups with the urinary bladder catheterised. Abdominal entry was through a midline incision, a peritoneal survey was performed and the splenic flexure of the colon was routinely taken down. The left ureter was identified and protected using a vascular sloop, the inferior mesenteric artery was ligated and divided just distal to the origin of the ascending left colic arterial branch and the inferior mesenteric vein was divided at the inferior border of the pancreas. Pre-sacral dissection was performed using a combination of sharp scissor dissection and electrocautery in a plane anterior to the hypogastric nerves preserving the entire mesorectal envelope in-tact down to the pelvic floor. In the case of ultra-low resection, dissection was continued inferiorly in the inter-sphincteric plane. Following anorectal transection and removal of the specimen of rectum with its tumour and mesorectal package, intestinal continuity was restored by tension-free stapled anastomosis. A proximal diverting loop ileostomy was performed in all, which was reversed at a median of four months after operation. The abdomen was closed using mass fascial closure with a pelvic drain in place.

Pathology specimens were fixed in a 10% formol-saline solution and processed in paraffin wax. Sections were stained in haematoxylin and eosin. The degree of tumour regression was graded according to the TRG grading system described by Mandard et al [22]. Based on this system of classification, TRG 1 was a complete response with the absence of residual cancer and fibrosis extending through the wall; TRG 2 was the presence of residual tumour cells scattered through the fibrosis; TRG 3 was an increase in the number of residual cancer cells compared with TRG 1, in which fibrosis was predominant; TRG 4 was residual cancer outgrowing fibrosis and TRG 5 was the absence of regressive changes. Furthermore, to facilitate analysis, the degree of TRG was categorised into subgroups; good regression – TRG 1 + 2; moderate regression – TRG 3; no regression – TRG 4 + 5. The reports of pathology specimens were reviewed at a bi-weekly clinico-pathology conference.

### Post-operative follow-up

Post-operative follow-up at the surgical clinic was at 2 and 4 weeks, and thereafter, 3 monthly for 2 years, 6 monthly for 3 years and annually thereafter. Follow-up comprised clinical examination, serum CEA testing 3 monthly for 3 years, 6 monthly at 3 to 5 years and annually thereafter. Colonoscopy and contrast-enhanced CT scan of the abdomen was undertaken at 1, 3 and 5 years after operation. Data from pre-operative, operative, post-operative and follow-up charts were entered into a prospectively collected colorectal database, which was maintained at the professorial surgical unit. Recurrence of cancer was identified by observation of either serial increase in serum CEA levels, followed by digital and endoscopic examination of the anastomosis with biopsy, which was followed by either computerised tomography alone or combined with positron emission tomography.

### Data management

Data were analysed retrospectively and presented as means with standard deviations (SD), medians with interquartile ranges (IQR) and frequencies with percentages (%). The difference between groups was evaluated using Pearson’s Chi-square, Mann Whitney U and Kruskal-Wallis tests as appropriate. Initially, a single variable analysis was performed to screen variables, following which, a multiple variable analysis was performed to determine an association between variables. Survival probabilities using Kaplan- Meier curves were calculated for different categories of TRG. Both overall survival (OS) and local recurrence-free (LRF) survival were analysed. All analyses were performed using the SAS system V 9.00, 2003 (SAS Institute, Cary, North Carolina, USA) and R open-source software. A P value of < 0.05 was considered significant. The study was approved by the Ethics Review Committee of the University of Kelaniya Medical School, based on a National Research Council Grant #00–25.

## Results

### Patient flow and follow-up

**Fig. 1:**
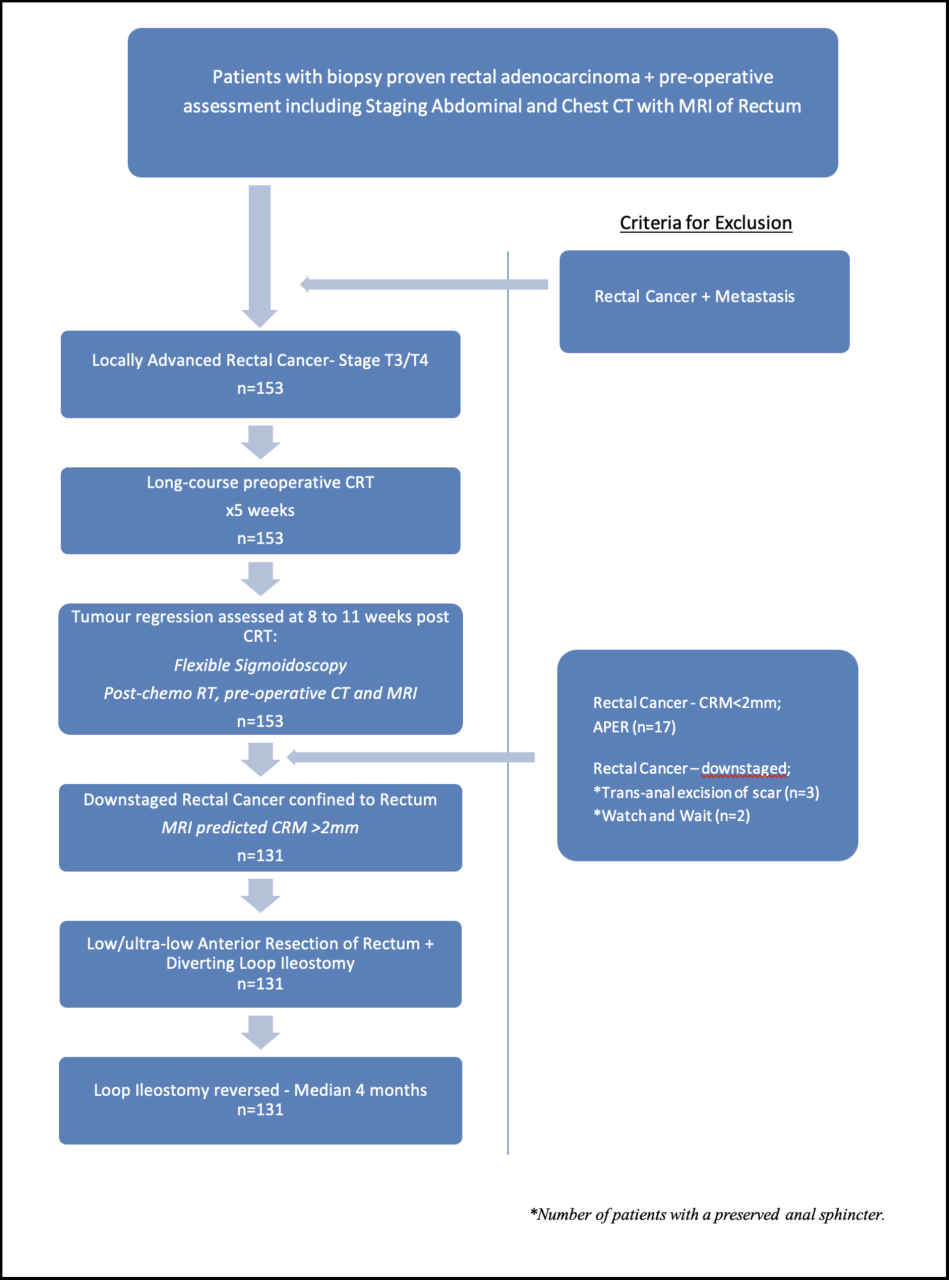
Flow-chart of patient management

Over fourteen years, 153 patients underwent pre-operative CRT for locally advanced rectal carcinoma at this single centre. Patients who underwent APER (n = 17), trans-anal resection (n = 3) and non-operative management with close follow-up (n = 2) were excluded from the analysis. Thus, the rate of anal sphincter preservation and avoidance of a permanent stoma was 89% (n = 136). In all, 131 (79 male, 52 female; median age 57, interquartile range 47–62 years) patients underwent low or ultra-low anterior resection of the rectum post-CRT – 4 (3%) developed anastomotic leakage. Twenty-two patients were excluded from the study after recruitment (Fig. 1). Median follow-up at the time of analysis was 15 months (interquartile range 6–45 months).

### Specimen Pathology and Tumour Regression

In our analysis of pathology reports of 131 resected rectal cancers, in 115 (88%), microscopy revealed a 2mm tumour-free CRM, with tumour-free proximal and a distal resection margin (> 10mm) - an R0 resection. In 16 rectal cancer specimens, the advancing tumour margin was within 1mm of the CRM – R1. Approximately 55.8% of patients showed either TRG 1 or TRG 2, that is, no evidence of tumour or a few scattered tumour cells (Table 1). In all, 78.4% of patients showed tumour regression (TRG 1 - TRG 3) compared to 21.6% who showed either minimal or no tumour regression (TRG 4 + 5). There was no statistically significant difference within the subgroups of responders to pre-operative chemoradiation; good (TRG 1 + 2), moderate (TRG 3) and poor (TRG 4 + 5) - (Chi-Square = 2.58; DF = 2; P = 0.28, Table 1). However, as expected, those with a good TRG had a median pathology T stage of 2 whereas those with poor TRG had a median pathology T stage of 3 (Chi-square 16.6; DF = 2; P = 0.0002).

**Table 1:** Percentage rectal cancer regression expressed in TRG grades

Regression grade	Percentage
TRG 1	43.1% (Good)
TRG 2	12.7% (Good)
TRG 3	22.6% (Moderate)
TRG 4 + 5	21.6% (Poor)

Good vs. Moderate vs. Poor - Chi-Square = 2.58; DF = 2; P = 0.28

The median number of lymph nodes harvested in TRG 1 + 2 + 3 (good and moderate regression) was 6 compared to the median number of 8 nodes that were harvested in the TRG 4 + 5 (poor regression; Chi-square 2.37; DF = 2; P = 0.31), which indicated comparable nodal harvest in these groups. Pathology nodal status in the sub-groups of TRG was evaluated in only 120 patients. In 11 patients, distributed evenly between good, moderate and poor regression groups, lymph node data were incomplete. As expected, those with poor TRG demonstrated, on average, higher pathological nodal status (pN1or N2 – at least one lymph node involved by cancer) compared with those who had good TRG (Chi-square 8.61; DF = 2; P = 0.0135 – Table 2). Furthermore, there was no significant difference between gender and the degree of TRG (Chi-square 0.0771; DF = 2; P = 0.96), age and TRG (Chi-square 3.83; DF = 2; P = 0.147) and between pre-operative CEA and TRG (Chi-square 0.653; DF = 2; P = 0.72).

**Table 2:** Pathological nodal status and the degree of TRG in 120 patients undergoing Anterior Resection

	N0	N1	N2	Total
Good regression (TRG 1 + 2)	33/120 (27.5%)	4/120 (3.33%)	7/120 (5.83%)	44
Moderate regression (TRG 3)	19/120 (15.83%)	7/120 (5.83%)	9/120 (7.5%)	35
Poor regression (TRG 4)	16/120 (13.33%)	14/120 (11.67%)	11/120 (9.17%)	41

**Fig. 2:**
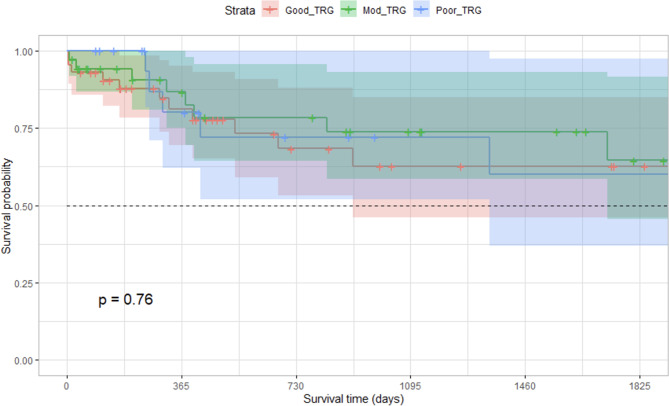
Comparison of overall survival between tumour regression grades

**Fig. 3:**
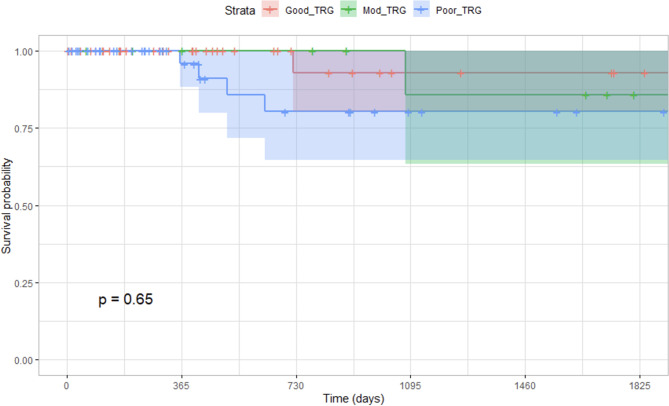
Local recurrence-free survival between tumour regression grades

Local recurrence was found in 6.8% of patients (9 of 131) undergoing anterior resection of the rectum and there was no significant difference in overall survival (Fig. 2) or disease-free survival (Fig. 3) amongst the different groups of tumour regression. The 5-year disease-free survival in good, moderate and poor TRG groups was 65%, 83% and 67% respectively (Chi-square − 0.01; DF – 2; P = 0.99). Overall survival in the poor TRG group was 65%, while in the good TRG group, it was 71% (Chi-square – 0.14; DF – 1; P = 0.7).

## Discussion

In Sri Lanka, cancer of the colon and rectum is now the third most frequent cancer for all ages and both genders, up from being the seventh most common cancer in 2008 [23, 24]. From a later study of colorectal cancer patients from the same district, more than two-thirds of cancers were found to lie within the rectum [25]. The management of rectal cancer is complex and requires a specialised multidisciplinary team comprising surgeons, radiologists, oncologists and pathologists, including nutritionists and enterostomal care teams. Data from South Asia regarding the treatment and outcome of rectal cancer in the era of pre-operative chemoradiation are sparse. This study presents a “snap-shot” of the results from a single specialised centre of pre-operative chemoradiation for selected patients with rectal cancer with a specific focus on the impact of CRT on tumour regression and local recurrence, disease-free survival and overall survival following open low anterior resection of the rectum for cancer. We did not include data of rectal cancers that were removed laparoscopically because the study was performed at a time of the learning curve for laparoscopic surgery for team members, which therefore had the potential to skew the result of pathology reporting. Also, at our centre, we chose to use long-course chemoradiation over the option of short-course chemoradiation because the waiting time for surgery after CRT in the former was longer compared with the latter. This supported waiting times for surgery in the department’s busy operative schedules and enabled better planning for patients.

In conclusion, our study has shown that the majority with rectal cancer who have either involved or threatened mesorectal margins on pre-operative assessment will have tumour regression following long-course chemoradiation, which will enable restorative rectal resection. A limitation of this study was the possibility of inter-observer variability among our pathologists, the suboptimal number of lymph nodes harvested by our pathology team and the relatively small number of patients. Even though it is possible to achieve the global benchmark of local recurrence for mid and low rectal cancers, predicted overall survival will remain unchanged with the use of current chemoradiation protocols. Total neoadjuvant therapy seems promising in its ability to improve longer disease-free survival.

## Data Availability

The dataset used and analysed for this study is available from the corresponding author upon reasonable request.

## List of abbreviations

CRT: Chemoradiotherapy
TRG: Tumour regression
TME: total mesorectal excision
CEA: carcinoembryonic antigen
AJCC: American Joint Committee on Cancer
CIM: Cancer Institute Maharagama
IQR: interquartile ranges
OS: overall survival
LRF: local recurrence-free
CRM: circumferential resection margin
APER: abdominoperineal excision of the rectum
